# Selective visceral perfusion in thoracoabdominal aortic surgery: optimal flow rate in a porcine model

**DOI:** 10.1007/s10047-025-01521-y

**Published:** 2025-07-30

**Authors:** Noburo Ohashi, Hajime Ichimura, Noritoshi Kikuchi, Yuki Tanaka, Tohru Mikoshiba, Yuko Wada, Kenji Okada, Tatsuichiro Seto

**Affiliations:** 1 Devision of Cardiovascular Surgery, Department of Surgery, Shinshu University School of Medicine, 3-1-1, Asahi, Matsumoto City, Nagano 390-8621 Japan; 2 Department of Clinical Engineering, Shinshu University Hospital, Matsumoto, Japan; 3 Interdisciplinary Cluster for Cutting Edge Research, Institute for Biomedical Science, Shinshu University, Matsumoto, Japan; 4 Department of Surgery, Division of Cardiovascular Surgery, Kobe University Graduate School of Medicine, Kobe, Japan

**Keywords:** Thoracoabdominal aortic surgery, Selective visceral perfusion, Cardiopulmonary bypass, Porcine model

## Abstract

To determine the optimal perfusion volume for the celiac artery (CA) and superior mesenteric artery (SMA) in a porcine model. Fifteen Yorkshire pigs (46.7 ± 5.2 kg) underwent selective CA and SMA perfusion using a roller pump at either 400 mL/min (G400, n = 5) or 800 mL/min (G800, n = 6). Hemodynamic parameters, blood gas analyses, and biochemical markers were evaluated over time (T1 to T5). The intestinal tissue was assessed for edema and histological damage. Portal vein SvO2 was lower in G400 (65.0 ± 30.2% at T2) compared to G800 (87.0 ± 5.2%), indicating reduced perfusion. Lactate levels were significantly higher in G400 (7.8 ± 2.3 mmol/L at T2) than in G800 (4.1 ± 2.1 mmol/L), suggesting increased anaerobic metabolism. Aspartate aminotransferase levels were elevated in G400, reflecting intestinal ischemia, whereas alanine aminotransferase levels remained stable. Histological analysis revealed mucosal desquamation in G400 but not in G800. No significant differences in intestinal edema were observed between groups. A selective perfusion volume of 800 mL/min for the CA and SMA maintains portal vein SvO2 and prevents mucosal injury, suggesting it approximates physiological blood flow. These findings indicate that increasing selective visceral perfusion during thoracoabdominal aortic surgery may reduce postoperative intestinal complications and improve patient outcomes.

## Introduction

Open repair of a thoracoabdominal aortic aneurysm remains a challenging procedure with various complications and a high mortality rate [1]. Complications related to postoperative outcomes have been reported, including spinal cord ischemia, cardiac events, stroke, respiratory complications, and renal failure requiring permanent dialysis. Other important factors include intestinal complications; however, their association with postoperative mortality remains controversial.

Many centers use selective organ perfusion with partial extracorporeal circulation during thoracoabdominal aortic aneurysm surgery [2–4]. Although selective perfusion of the celiac artery (CA) and superior mesenteric artery (SMA) has been found to reduce the incidence of intestinal complications in pigs [5, 6], few clinical studies have described the actual perfusion volumes to the CA and SMA. In addition, there are no reports of in vivo experiments examining the optimal perfusion volume.

Therefore, this study aimed to determine the optimal perfusion volume for the CA and SMA in a porcine model.

## Materials and methods

### Animal surgery

This study was conducted in accordance with the ARRIVE (Animal Research: Reporting of In Vivo Experiments) guidelines for the ethical reporting of animal research. The experimental protocol was approved by the Committee for Animal Experiments of Shinshu University and adhered to the national regulations and guidelines for animal experiments in Japan. All efforts were made to minimize animal suffering and to use the minimum number of animals necessary to achieve scientific validity.

Fifteen female Yorkshire crossbreed pigs (46.7 ± 5.2 kg) underwent selective CA and SMA perfusion using a roller pump at either 400 mL/min (G400) or 800 mL/min (G800). The surgical procedure was performed while maintaining a normal rectal temperature. Pigs were premedicated intramuscularly with atropine (0.5 mg), xylazine (0.1 ml/kg), and ketamine (0.1 ml/kg). Anesthesia was induced intravenously using pentobarbital (25–30 mg/kg) and vecuronium bromide (0.5 mg/kg), followed by endotracheal intubation. Anesthesia was maintained by inhalation of 1.5% isoflurane in an air mixture with 100% oxygen using intermittent positive-pressure ventilation support (PRO-45Va Acoma Medical, Tokyo, Japan).

The pigs were fixed in the supine position, and we measured the arterial systemic blood pressure (from the femoral artery) and peripheral oxygen saturation (SpO2) and performed an electrocardiogram. Extracellular fluid infusion was used for intraoperative maintenance, and dopamine was used when the blood pressure decreased.

**Fig. 1:**
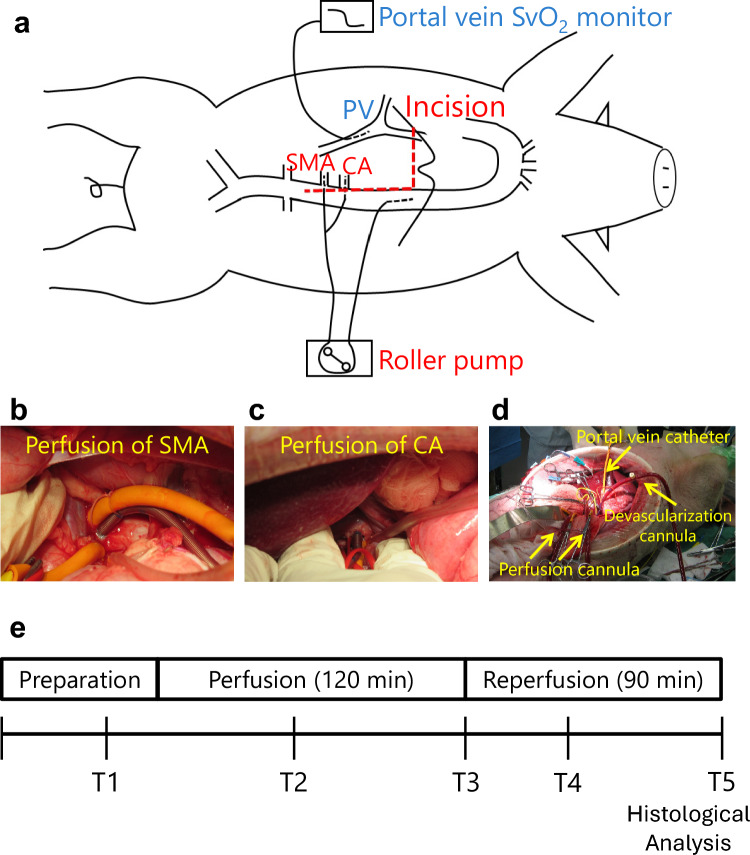
**a** Surgical schematic. Devascularization from the descending aorta and selective perfusion of SMA (**b**) and CA (**c**) with a 10 Fr arterial cannula. An SvO2 measurement catheter was placed in the portal vein (**d**) SvO2 venous oxygen saturation; *SMA* superior mesenteric artery, *CA* celiac artery

The abdomen was opened through an epigastric incision, as shown in Fig. 1. The portal vein was dissected, and a catheter for venous oxygen saturation (SvO2) measurement (Edwards oximetry CV catheter, USA) was inserted into the portal vein. The CA and SMA were taped, and heparin (300 U/kg) was intravenously administered to maintain the activated clotting time (ACT) above 400 s. A 14 Fr blood removal cannula (Medtronic No.77014, USA) was inserted into the descending aorta, and a 10 Fr arterial cannula (Medtronic one-piece pediatric arterial cannula No.77010, USA) was punctured in the aorta and selectively inserted into the CA and SMA. The perfusion flow rate was set, and selective perfusion was initiated using a roller pump (HAS-P100, Izumiko Medical Industry Japan). After 120 min of perfusion, the catheter was removed and reperfused, followed by 90 min of observation (Fig. 1).

### Blood test

Blood samples were obtained at T1 before selective visceral perfusion, T2 60 min after perfusion, T3 120 min after perfusion, T4 30 min after reperfusion, and T5 90 min after reperfusion. Arterial potential hydrogen (pH), oxygen pressure (PaO2), base excess (BE), lactate (Lac), aspartate aminotransferase (AST), alanine aminotransferase (ALT), creatine kinase (CK), and lactate dehydrogenase (LDH) levels were measured (Fig. 1).

### Tissue examination

After 90 min of reperfusion, the pigs were sacrificed by intravenous thiamylal administration. Their intestinal tracts were removed; some were fixed using paraformaldehyde, and the rest were used for weight determination. The fixed organs were embedded in paraffin, sliced into 4 μm slices, and used for tissue analysis.

We performed histological tests (hematoxylin and eosin; H&E) and assessed the dry and wet weights of the jejunum. The intestinal wall was excised at T5 and histologically evaluated. The dry–wet weight ratio was also measured to evaluate tissue edema by leaving the intestinal tissue in a constant temperature bath at a drying temperature of 105 °C for 16 h and measuring each weight using a precision electronic balance (n = 4 at G400 and n = 5 at G800). It was calculated as follows:

(weight before drying / weight before drying) × 100 (%).

### Statistical analysis

Differences between the two groups were compared using the repeated functional ANOVA in Statistical Package for the Social Sciences (SPSS, version 23). For the dry–wet weight ratio, a t-test was performed to examine differences between the two groups.

## Results

### Pilot study for groupings

**Fig. 2:**
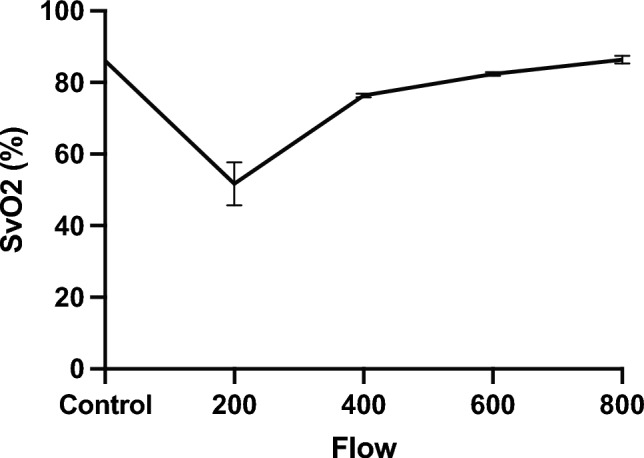
Changes in portal vein SvO2 associated with changes in CA and SMA perfusion over time SvO2 venous oxygen saturation; *SMA* superior mesenteric artery, *CA* celiac artery

First, a pilot study was conducted to identify groups. The physiological flow rates of the human CA and SMA are approximately 400 mL/min, and in pigs, it is approximately 1.5 times higher. Therefore, as a pilot study, SvO2 values were measured in one pig when the CA and SMA perfusion volumes were changed in steps of 200–800 mL/min (Fig. 2). The SvO2 was measured thrice at 200, 400, 600, and 800 mL/min before perfusion. The SvO2 increased with increasing perfusion volume, and at 800 mL/min, SvO2 was 86.3 ± 1.1%, increasing to the same level as before perfusion (86.0 ± 0.0%) (Fig. 2). Since an extreme decrease in SvO2 was observed at a perfusion volume of 200 mL/min (76.3 ± 0.6%), and since this flow rate is considerably lower than that reported in clinical papers, we decided to compare two groups in this study: one group perfused at 400 mL/min (G400) and the other group perfused at 800 mL/min (G800).

### Animal characteristics

Eight pigs were included in the G400 group and six in the G800 group; two of the pigs in the G400 group died during the experiment, and one of the G400 animals was excluded due to insufficient data collection. Of the two animals that died, one died immediately after the start of pumping, possibly due to the surgical technique, and the other died after reperfusion due to uncontrolled bleeding. Therefore, data were collected and analyzed using five pigs for G400 and six pigs for G800. At the beginning of the experiment, body weight was 44.8 ± 0.4 kg for G400 and 48.5 ± 6.8 kg for G800; heart rate was 116 ± 22 beats/min and 97 ± 11 beats/min; systolic blood pressure was 123 ± 26 mmHg and 108 ± 22 mmHg; SvO2 was 84.7 ± 7.6% and 87.0 ± 6.2%. None of the differences were significant.

### Hemodynamics after perfusion

**Fig. 3:**
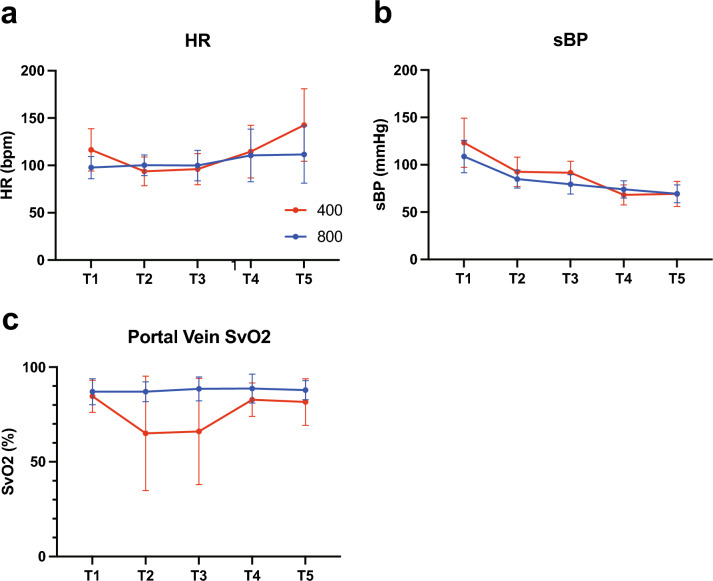
Changes in the heart rate (**a**), blood pressure (**b**), and SvO2 (**c**) over time in G400 and G800 SvO2 venous oxygen saturation

The changes in heart rate, systolic blood pressure, and SvO2 during the experiment are shown in Fig. 3. Heart rate and systolic blood pressure did not differ between the two groups. SvO2 decreased by 65.0 ± 30.2% and 66.0 ± 28.1% at T2 and T3 in G400, respectively; however, there was no statistically significant difference when compared to G800 (87.0 ± 5.2% at T2 and 88.5 ± 6.3% at T3).

### Blood gas analysis

**Fig. 4:**
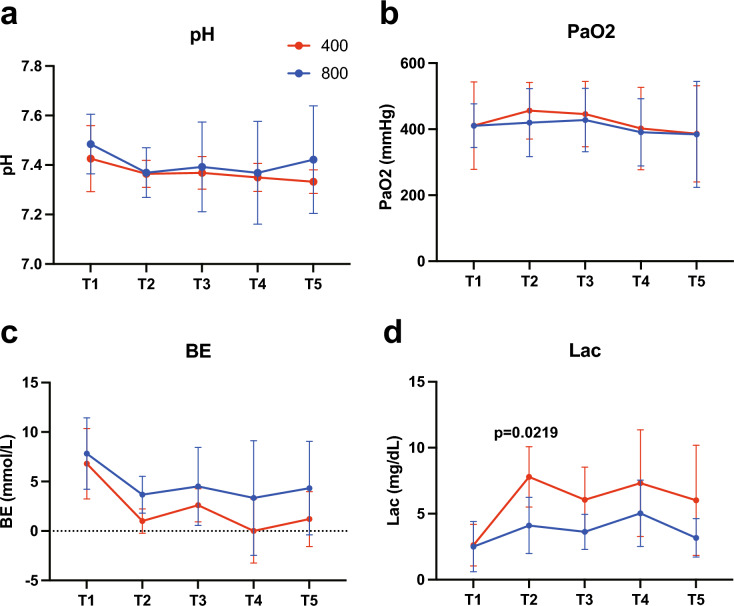
Changes in pH (**a**), PaO2 (**b**), BE (**c**), and Lac(**d**) over time in G400 and G800 in the arterial blood gas test PaO2 arterial oxygen pressure, *BE* base excess, *Lac* lactate

The arterial blood gas test results are shown in Fig. 4. The pH and PaO2 did not change significantly during the experiment in either group. BE tended to be lower in G400 at T2 (1.0 ± 1.2 mmol/L) than in G800 (3.7 ± 1.9 mmol/L). Lac tended to be higher in G400 from T2 to T5, with a value of 7.8 ± 2.3 mmol/L at T2, which was significantly higher than that of G800 (4.1 ± 2.1 mmol/L) (p = 0.0219).

### Blood biochemistry analysis

**Fig. 5:**
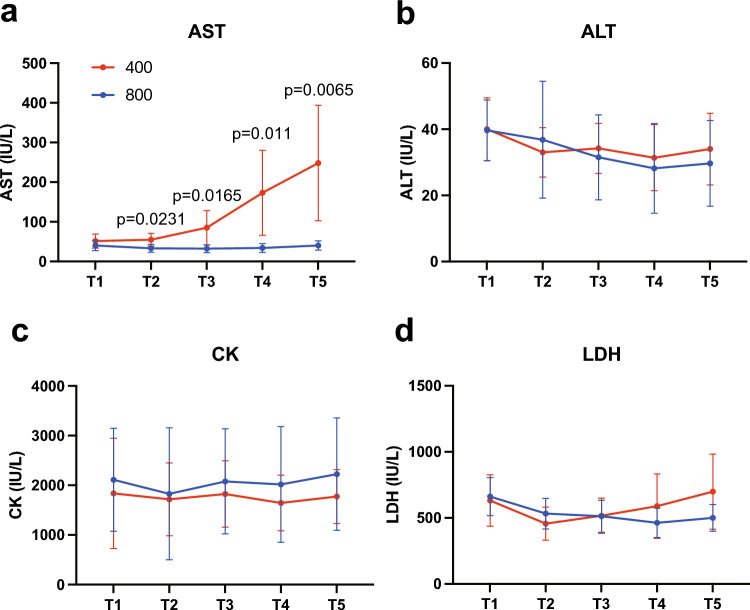
Changes in AST (**a**), ALT (**b**), CK (**c**), and LDH (**d**) over time in G400 and G800 in the arterial blood gas test *AST* aspartate aminotransferase, *ALT* alanine aminotransferase, *CK* creatin kinase, *LDH* lactate dehydrogenase

Figure 5 shows the results of the arterial blood biochemistry tests. CK and ALT were generally constant throughout the experiment, while AST and LDH showed an increasing trend over time in G400. In particular, AST was significantly elevated at T2 (54.6 ± 16.0 IU/L, p = 0.0231), T3 (85.0 ± 43.2 IU/L, p = 0.0165), T4 (173.0 ± 107.4 IU/L, p = 0.011), and T5 (248.2 ± 145.7 IU/L, p = 0.0065) compared to G800. LDH showed an increasing trend in G400; there was an increase in G400 (699.0 ± 284.6 U/L) and G800 (500.3 ± 101.4 U/L) at T5, but the difference was not statistically significant.

### Investigation of intestinal edema

**Fig. 6:**
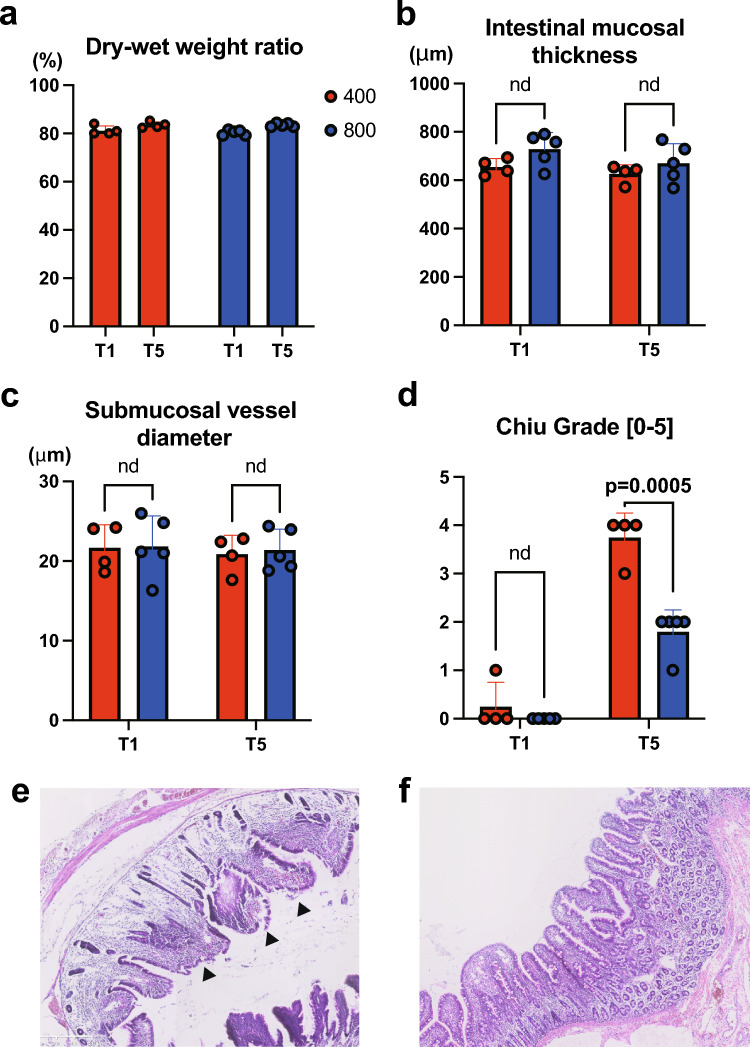
**a** The dry–wet weight ratio of the small intestine excised during autopsy. **b-d** Intestinal mucosal thickness (**b**), Submucosal vessel diameter (**c**) and the grade of mucosal damage by Chiu scoring system (**d**) compared between G400 and G800 at T1 and T5. Hematoxylin and eosin (H&E) staining of the small intestine in G400 (**e**) and G800 (**f**). The arrowhead in (**e**) indicates Chiu grade 4 endometrial damage

The weight of the intestine removed before perfusion and at the end of the experiment, as well as the weight after drying, was measured, and the dry–wet weight ratio was calculated. The weight of G400 was 81.28 ± 0.02% before perfusion and 83.52 ± 0.01% at the end of the experiment, which did not differ from that of G800 (80.34 ± 0.01% and 83.48 ± 0.01%). No obvious findings of intestinal edema were observed (Fig. 6a).

### Histological analysis

A portion of the excised small intestine was paraffin-embedded and histologically confirmed by H&E staining for stromal edema and mucosal desquamation. Mucosal desquamation was observed in the G400 group but not in the G800 group (Fig. 6e and f). The present study employed the Chiu scoring system to quantify the extent of intestinal mucosal damage. The results revealed a significant increase in the Chiu grade at T5 [7]. Moreover, G400 demonstrated a significantly higher Chiu grade than G800 (Fig. 6d). Furthermore, the potential for edema to be caused by ischemia at G400 and overperfusion at G800 was subjected to histological evaluation. The widening of the villus tip–mucosal muscle plate distance (Fig. 6b) and the presence or absence of vascular dilation in the submucosal layer (Fig. 6c) were quantitatively assessed; however, no significant differences were observed between T1 and T5 in either group.

Moreover, no bleeding associated with overperfusion was observed at G800.

## Discussion

During surgical intervention on the thoracoabdominal aorta, securing blood flow in the CA and SMA is an important issue for intestinal protection [1, 8, 9]. While the incidence of intestinal ischemia during thoracoabdominal aortic surgery is relatively low, the occurrence of severe complications is notably high in cases where it does arise. Achouh et al. reported that intestinal ischemia was a risk factor for perioperative mortality during surgery in 1159 patients with a thoracoabdominal aortic aneurysm [4]. However, most perfusion volumes reported in previous clinical studies during selective perfusion of the intestinal tract are < 200 mL/min [2, 3], which is only approximately half to a quarter of the 400 to 800 mL/min flow rate considered to be physiological blood flow in the CA and SMA in humans [10, 11]. This phenomenon can be attributed to the fact that the flow rate to the branches is determined by the circuit pressure allowed by the fluid delivery characteristics of the cannula used. For instance, the cannulas employed during surgical procedures (Kawasumi, Sumius 10 Fr or 12 Fr) exhibit a maximum CA and SMA flow rate of 450 mL/min at a circuit pressure of 250 mmHg, which precludes the attainment of higher flow rates. Therefore, due to technical limitations, the blood flow rate to the CA and SMA is restricted, making it challenging to determine the optimal blood flow rate for protecting abdominal organs during extracorporeal circulation in clinical studies.

It is acknowledged that pigs possess biological functions analogous to those observed in humans. Consequently, they have frequently been used as models to simulate the effects on humans, particularly in the context of cardiopulmonary bypass [12]. A perfusion model of the thoracoabdominal aorta in pigs was first reported by Kalder et al. in 2012, in which they performed selective perfusion at 200 mL/min to the CA and 400 mL/min to the SMA and reported less intestinal mucosal damage in the selective perfusion group than in the simple blockade of the thoracic aorta [5]. The same group subsequently reported that Lac levels were elevated and acidosis progressed in the group that underwent the same selective perfusion as above, compared with the group that underwent simple blockade and perfusion to the aorta distal to that blockade [13]. In a similar experiment, they observed the progression of intestinal apoptosis in simple blockade and selective perfusion groups [6]. They compared a control group without thoracic aortic cross-clamping (TAC); a group with TAC; TAC and distal aortic perfusion (DAP); and TAC, DAP, and selective visceral perfusion (SVP). The results suggested that damage to the intestinal mucosa was greater in the SVP group than in the DAP-only group. However, in this study, the perfusion pumps for DAP and SVP were the same, and the flow rate for SVP was not set; therefore, it was unclear whether the optimal SVP was used. Based on these results, it can be concluded that the abovementioned perfusion volumes in pigs are insufficient to protect the intestinal mucosa; however, the ideal visceral artery perfusion rate is unclear.

Therefore, we conducted this study to determine the optimal amount of selective perfusion in the intestinal tract of pigs. In a previous study, the physiological blood flow rates in pigs were reported to be 600 mL/min for the CA and 1000 mL/min for the SMA [5]. The perfusion volumes of 400 and 800 mL/min used in this experiment can be considered to be approximately half of the physiological blood flow rate and the same perfusion volume as the physiological blood flow rate. In other words, this comparison can be interpreted as comparing the effect of a perfusion volume commonly used in previous reports on humans (approximately 200 mL/min) with that of a volume approximately equal to physiological blood flow (approximately 400 mL/min), which is approximately double the volume.

In this study, we found a decrease in the SvO2 and an increase in Lac during perfusion at G400, similar to the findings in previous reports of decreased perfusion of abdominal organs. In addition, our experiments showed a significant increase in AST levels from T2 to T5 but no increase in ALT levels. These findings indicate a reduction in SvO2 at T2 and T3, which could be attributed to either intestinal or liver blood flow. Regarding SvO2 in G400, the standard deviation became very large. There were no apparent differences between the experimental subjects in terms of pre-operative status, measurement techniques, and surgical techniques, and the cause of the variation in measurement values is unclear. However, the variation occurred only in the group with low blood flow, and it cannot be ruled out that there were individual differences. Since previous reports have confirmed that Lac and AST levels are predominantly elevated in the acute phase of patients with intestinal ischemia, and no elevation in ALT was observed, it can be inferred that the changes in blood chemistry tests in the present study reflect intestinal ischemia [14, 15]. Zogheib et al. reported that the AST, Lac, and myoglobin levels were significantly elevated in patients with bowel ischemia after cardiac surgery compared to the non-ischemic group, and they proposed a scoring system that can be used to detect intestinal ischemia at an early stage, reporting its usefulness [15].

In the histological analysis, although edema of the intestinal tract was not evident, intestinal mucosal desquamation was observed in G400, which is consistent with the findings presented above. In contrast, the post-perfusion changes observed in G400 were not observed in G800, suggesting that the abdominal organs were protected by a perfusion volume of 800 mL/min, which was close to the physiological blood flow rate in pigs. No previous porcine experiments have reported perfusion volumes of 800 mL/min in the CA and SMA.

In addition, the elevated liver transaminase levels observed in this study may reflect liver damage. In terms of histologic units, the liver lobule is divided into the portal zone (zone 1), the intermediate zone (zone 2), and the central zone (zone 3). Zones 1 and 2 have a rich blood flow of oxygen and nutrients, whereas zone 3 has a low partial pressure of oxygen. AST > ALT levels were measured in patients with hypoxic liver injury due to circulatory failure. In this study, we also observed a significant increase in AST levels in the G400 group but no increase in ALT levels. This suggests that G400 may cause ischemic liver damage owing to low blood flow [16].

Our results suggest that a selective perfusion volume of 400 mL/min to the CA and SMA during surgery to the thoracoabdominal aorta, which is approximately twice the 200 mL/min perfusion volume currently used in practice, may be essentially required; however, a higher flow rate is desirable if possible. An increased perfusion volume is expected to reduce postoperative intestinal or liver complications, thereby improving postoperative outcomes. However, as previously mentioned, the cannulas currently used in clinical procedures have a maximum irrigation flow rate of approximately 450 mL/min due to their fluid delivery characteristics [17]. In the present study, a one-piece cannula with optimal fluid delivery characteristics was employed to attain a sufficient irrigation flow rate. However, this cannula was found to be incompatible with abdominal branch perfusion. Consequently, there is a necessity for the advancement of cannulas with enhanced fluid delivery characteristics.

This study has several limitations. First, the perfusion flow rate was set based on the pump output, but the actual flow rates in the celiac artery (CA) and superior mesenteric artery (SMA) were not measured. As a result, the extent of flow reduction due to pressure loss remains unclear. Furthermore, since the flow rate to each organ perfused by each artery was not measured, it remains unclear whether the phenomenon of ischemia actually occurred in the intestines and liver. However, in clinical practice, the blood flow to the CA and SMA is predicted only by the blood delivery volume and pressure on the pump side. We believe that the model used in this study is appropriate for simulating problems that occur in clinical practice. Second, we were not able to assess the effects on other abdominal organs, such as the liver. Elevated levels of AST are also frequently observed in liver disorders. However, since the histological examination in this study did not rule out liver disorders, it remains unclear whether the observed increase in AST levels is solely due to intestinal injury. Third, in this study, surgical procedures, such as arterial occlusion and vascular anastomosis, were not performed during perfusion; therefore, the experiment did not reflect actual surgical conditions. As a result, the effects of perfusion flow rate on surgical procedures, such as changes in blood loss resulting from variations in perfusion flow rate, could not be evaluated. Additionally, anesthesia methods commonly used in clinical practice, such as hypothermic anesthesia or intravenous anesthesia with propofol, were not employed. Therefore, strictly speaking, this model does not accurately simulate the conditions of clinical surgery. Moreover, this study did not include a control group that underwent a sham surgical procedure. Finally, given the limited number of samples from large animals included in this study, further consideration is necessary to determine whether SvO2 alone can be used as an indicator of organ damage or whether the development of a scoring system incorporating additional indicators is warranted. Further research is needed to address these limitations.

## Conclusion

This is the first study to successfully maintain the SvO2 in the portal vein and prevent intestinal mucosal desquamation using a perfusion volume that approximates the physiological blood flow rate of the abdominal artery in pigs. In humans, achieving a perfusion volume approximately twice the current level may further reduce intestinal complications during thoracoabdominal aortic surgery.

## Data Availability

The data that support the findings of this study are available from the corresponding author upon reasonable request.
